# Orthopaedics and Additive Manufacturing: The Start of a New Era

**DOI:** 10.12669/pjms.38.3.5182

**Published:** 2022

**Authors:** Hisham Khan Gandapur, M. Suhail Amin

**Affiliations:** 1 Dr. Hisham Khan Gandapur, MBBS. Postgraduate Resident Orthopaedics; 2 Prof. Maj. Gen. M. Suhail Amin, MRCS (Ed), MCPS (HPE), FCPS (Surg), FCPS (Ortho). Dean, Armed Forces Postgraduate Medical Institute, Professor, Army Medical College, Rawalpindi, Combined Military Hospital, Rawalpindi, Pakistan

**Keywords:** Additive Manufacturing, Three-dimensional, Printing, Orthopaedics, Bioprinting

## Abstract

The aim of this article is to report the recent surge in use of additive manufacturing (AM) or three-dimensional printing (3DP) services in healthcare, especially the field of orthopaedics. Pakistan’s healthcare infrastructure has been slow in adapting and implementing this new technology which is an integral part of the industry 4.0. Various sources including Pubmed, ScienceDirect, Google Scholar and Google were utilised from June to august 2021 to extract articles and information on advantages of AM in orthopaedics. Furthermore, its possible acquisition by a hospital, educational or an industrial setup is also highlighted in this review.

## INTRODUCTION

Three-dimensional printing (3DP) or additive manufacturing (AM) or rapid prototyping is a relatively new technology that has recently gained popularity in trauma and orthopaedics. Volumetric digital imaging and communications in medicine (DICOM) computed tomography (CT) or magnetic resonance imaging (MRI) data are used to print precise fracture models and patient-specific guides with new affordable desktop 3D printers. These models improve surgeon’s understanding of anatomy and pathology through tactile and visual experience. Evidence has also revealed the impact of 3DP technology in reducing surgery time and blood loss.^1^

Furthermore, the development of metal additive manufacturing for patient-specific implants and prostheses is the most important and valuable addition in the field of orthopaedics.^2^ We intend to explore the basics of additive manufacturing and its published benefits in orthopaedics along with its possible penetration and prospects in the Pakistani healthcare sector.

### Printing Techniques

Data of a medical image (CT, MRI, others) is acquired in DICOM format, which is converted into a 3D model using computer-aided design (CAD) program and stored in a STL (Standard Tessellation Language) format. The STL file is a universal language that is read by all 3D printers. The quality of the printed object or model depends on the resolution of the medical image. High-resolution CT images are ideal for this purpose.^3^

Various printing techniques are used by different 3D printers that range from stereolithography apparatus (SLA), selective laser sintering (SLS), direct metal laser sintering (DMLS), electron beam melting (EBM), fused deposition modelling (FDM) to ultraviolet (UV) having unique characteristics and applications. However, the technology is mainly based on 2D slicing of a STL model and printing the 3D model by adding layers of a given material on top of each other, hence the name additive manufacturing.^4^

### In-hospital 3D Printing

Medical 3DP is proving to be a new diagnostic imaging tool that increases understanding and knowledge of morbid anatomy and leads to an optimal surgical approach.^5^ This potential positive impact on patient outcomes and personalized care has led to the wide adoption of 3DP facilities in the clinics, reducing the reliance on external sources. This has been referred to as the point-of-care (POC) 3DP and requires skilled manpower and close collaboration with radiologists.^6^ POC 3DP does not replace the regular factories but rather works side by side with translational research, teaching, and clinical innovation.^7^ Ballard et al. in their literature-based financial analysis, suggested that the cost savings through enhanced preoperative planning and reduction in OR time could be substantial and may make up for the cost to maintain a 3DP lab.^8^

**Fig.1:**
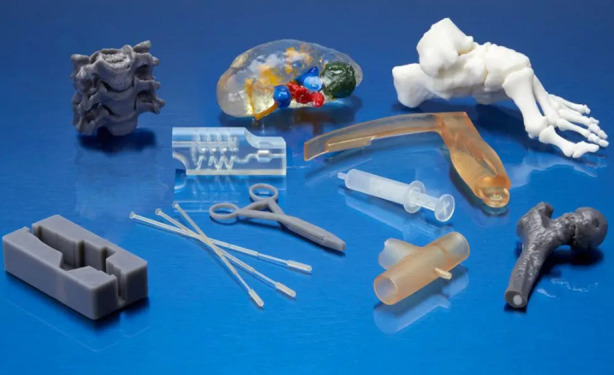
Biocompatible devices and anatomical models printed on Formlabs SLA 3D printer (Courtesy www.formlabs.com).^10^

The most common technology used for POC 3DP are SLA, FDM, and polyjet. SLA is the earliest 3DP technique first patented by Chuck Hull in 1986. 9 SLA uses highly precise laser light to cure liquid resins into plastic in a process known as photopolymerization. Currently, SLA models are most accurate, sterilizable and can be used during surgery for reference. A wide range of photocurable resins are available in opaque white and translucent consistencies. With the addition of medical grade biomed resins, SLA printers can also print biocompatible parts and medical devices for short to long term skin and mucosal membrane contact.^10^ Fig.1

The FDM technology uses thermoplastic filaments, such as ABS (Acrylonitrile Butadiene Styrene) and PLA (Polylactic Acid) that are melted and extruded from a heated nozzle on a build platform in a layer-by-layer technique. The FDM plastic models are not as accurate as their SLA counterparts and require a longer time to print in order to achieve comparable results. However, due to its larger build volume and low cost, FDM is preferred for simple design and education purposes. A high-performance thermoplastic like PEEK (Polyether ether ketone) has found its way in orthopaedics and traumatology due to its biocompatibility, low wear resistance and physical properties matching human bone.^11^ Honigman et al. found promising results in using medical grade PEEK biomaterial for the production of patient-specific implants inside a hospital setup.^12^ The radiolucent properties of PEEK implants have also been found beneficial in tumour surgery with fewer metal artefacts on follow up imaging and less beam scattering and attenuation in adjuvant radiotherapy.^13^ Other biocompatible materials (like ULTEM 1010) used to print patient-specific guides for total knee arthroplasty provide equivalent accuracy to metal instruments, with the added benefit of time and theatre cost savings during the procedure.^14^ Both FDM and SLA 3D printers were used extensively during the supply chain disruption in recent COVID-19 pandemic and provided the much needed life-saving medical devices in a short period of time.^15^

The polyjet printers use material jetting (MJ) technology and are the most versatile 3D printers, enabling multimaterial coloured anatomical models that mimic human bone and soft tissue.^16^ The new Stratasys J750 Digital Anatomy is a high-cost polyjet 3D printer that creates the most lifelike models available.^17^ This technique has expanded the applications of 3D digital printing and provides a remarkable design choices in the area of clinical research and education.^18^

### Education and Training

Many hospitals and institutions are beginning to use 3D printed anatomical models for teaching and training purposes, which have proved to increase learning effectiveness and cost-saving. Patient safety may also enhance as surgeons in training get the chance to perform on rare patient-specific scenarios before getting hands on real patients.^3^

**Table I:** Overview of common AM processes used in healthcare.

Printers in Use	Printing Process	Materials Used	Manufacturers	Common Applications	Estimated Cost of Machine (US$)	Material Cost (US$/Kg)	References
SLA	VAT Photopolymerization (VP)	Varieties of resin (thermosetting plastics). Standard, engineering (ABS-like, PP-like, flexible, heat-resistant), castable, dental, and medical (biocompatible)	FormLabs, 3D Systems	Medical Models, Splints & proethetics, Surgical guides	$3,000 (desktop)->$80,000 (industrial)	$149-$200/L of resins	10, 29
SLS	Directed Energy Deposition	Engineering thermoplastics, such as nylon	FormLabs, 3D Systems	Medical devices and tools	$10,000 (desktop), $100,000 (industrial)	$100/Kg for nylon	10, 29
SLM or DMLS	Directed Energy Deposition	Metal Powders (Ti6Al4V, Co-Cr-Mo, Al2O3-ZrO2)	EOS Group, SLM-Solutions	Implants	>$350,000	>$300/Kg	41
EBM	Powder Bed Fusion	Metal Powders (Ti6Al4V, Co-Cr-Mo, Al2O3-ZrO2)	Arcam, GE Additive, Qbeam	Implants, Surgical instruments	>$250,000	>$300/Kg	29,41
FDM/ FFF	Material Extrusion	Standard thermoplastics, such as ABS (Acrylonitrile Butadiene Styrene), PLA (Polylactic Acid), and their various blends	Stratasys, FormLabs	Medical Models, Splints & prosthetics, Surgical guides	$1,000->$15,000	$50-$150/Kg	10
ColorJet	Binder Jetting	Gypsum Based Powders (ZP150, ZP151), Metal powders	ExOne, Zcorp, 3D Systems, HP	Medical Models	$30,000 (desktop) - $450,000 (industrial)	-	29, 42
Polyjet	Material Jetting	Liquid (VeroWhite, VeroClear, TangoPlus, Multi-material)	Stratasys, Projet, (3D Systems), Polaris	Medical Models (Combination of multiple colours and materials)	$10,000 – >$250,000	$300-$1000/Kg	29

***Note:*** SLA (Stereolithography), SLS (Selective Laser Sintering), SLM (Selective Laser Melting), DMLS (Direct Metal Laser Sintering), EBM (Electron Beam Melting), FDM (Fused Deposition Modeling), FFF (Fused Filament Fabrication), Titanium- 6-Aluminum-4-Vanadium (Ti6A14V), Cobalt-chromium-molebdenum (Co-Cr-Mo), Aluminum-Zirconia (Al2O3-ZrO2).

Medical students benefit from the variety of 3D printed models with different pathologies compared to the not so readily available cadaveric material.^19^ Zhen et al. conducted a literature review and meta-analysis on the role of 3D printed models in teaching human anatomy and reported that students performed better in the 3DP group than the conventional group in terms of test scores, accuracy and student satisfaction.^20^

In addition, 3DP has been an excellent tool to aid in the informed consent process with patients. 3D printed models give patients a better understanding of their disease and treatment plan in a personalized manner and improve patients overall satisfaction.^21^

### Metal Additive Manufacturing

The 3DP methods used to manufacture metal implants are based on powder bed fusion (PBF) technology, where the source of energy is in the form of a laser or electron beam that selectively melts layers of metal powder bed.^22^ The cost of metal AM is higher than the conventional or subtractive manufacturing methods when mass production is required. However, metal AM has a potential for mass customization and producing complex geometries tailored to each patient’s anatomy.^23^

Electron beam melting (EBM) is one of the most common 3DP techniques applied for bio-metallic devices. It was first commercialised by a Swedish company named Arcam AB in 1997 and was recently acquired by General Electric (GE Additive, USA).^24^ EBM is a unique hotbed AM process working in a high vacuum to ensure a clean and controlled environment. This significantly reduces the thermal residual stresses in the parts and prevents oxidation of the metal compared to the selective laser melting (SLM) technique.^24^-^26^ In addition, the electron beam penetrates deep into the material with high absorption efficiency, resulting in lower power consumption.^27^ This also allows the use of larger powder particles and thicker layers to be built, which in turn leads to cost savings of up to 50%.^24^

**Fig.2:**
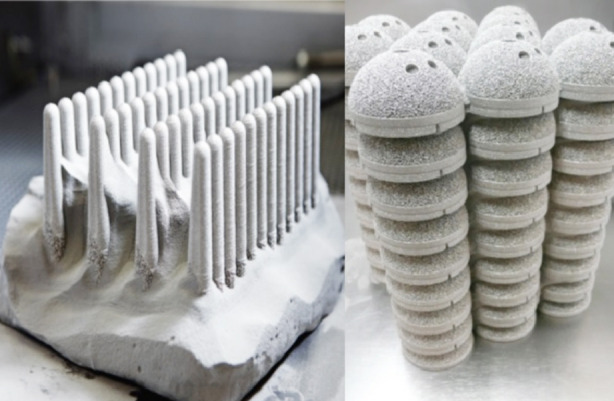
Parts stacked in sintered powder (Courtesy GE Additive).^28^

EBM is an industrial-grade machine and is increasingly being used for volume production. The ability to tightly stack parts together in a sintered powder leads to high productivity and cost savings per part (Fig.2).^28^ GE Additive’s new Arcam Q10 is a third-generation metal 3D printer available for orthopaedics implant manufacturers. Its price exceeds $250,000 USD^29^ and the company is providing training to end-users at various locations in Europe and the US, which further adds to the cost of acquiring this technology.

Titanium and its alloys (esp. Ti-6Al-4V) are most suited to EBM applications due to its brittleness and reactive properties.^30^ The freedom to design advanced trabecular structures on highly porous surfaces of 3D printed titanium implants (3DPTI) facilitates bony ingrowth and provide fixation stability without compromising the mechanical strength.^31^ Various functionalization procedures, through surface modification and drug or cell loading, of 3DPTI exist to enhance osseointegration and prevent infection.^32^ Recently, there has been a surge in hip cases using customized 3D printed acetabular components for varying degrees of acetabular defects with satisfactory mid-term radiological outcomes.^33^ Furthermore, a mid-term follow-up registry study on a large population comparing EBM-built titanium femoral stems to a cohort of traditional cementless stems reported no additional risks of aseptic loosening or mechanical failure.^34^

In addition to titanium, cobalt-chrome alloy (Co-Cr-Mo) and stainless steel (mainly 316L) are also applicable to AM by EBM or SLM methods. The cost of these powdered alloys is less than the titanium alloy. An Italian company, named REJOINT, is employing an innovative approach through artificial intelligence (AI), blockchain technology, and AM to customize cobalt-chrome knee prostheses and claims higher patient satisfaction and surgeon support.^35^

### 3D Bioprinting

3DP has geared up to include bioprinters that produce 3D matrices containing cells, substrates and growth factors in several combinations. Certain existing 3DP technologies are used alone or in combination, such as inkjet, stereolithography (SLA), laser-assisted and extrusion-based for bioprinting strategies.^36^ Inkjet is a droplet-based bioprinter and is commonly being used for producing functional tissue constructs.

Attempts are underway to regenerate bone and cartilage tissue with the help of 3D bioprinting.^37^ Both natural and synthetic bioprintable hydrogels have been developed acting as extracellular matrices (ECMs) that encapsulate osteoblasts and chondrocytes.^38^ Additionally, other nanomaterials, drugs and cytokines can also be incorporated into hydrogels to produce functional bone tissue scaffolds.^39^ Despite the success in vivo and in vitro studies, the gap between 3D bioprinting of transplantable tissues and its clinical applications still exist, and currently it seems challenging to meet the ethical and regulatory criteria.

### Scope of Medical 3DP in Pakistan

With the restrictions on the import and use of 3D printers in Pakistan, the country is lagging behind the world of the fourth industrial revolution, or industry 4.0.^40^ Although it is possible to get a no-objection certificate (NOC) from the Ministry of Interior, it is time-consuming and requires security clearance from several government organizations. Nonetheless, the 3DP industry has been evolving in recent years and some local manufacturers are playing a key role in breaking the barriers. These locally made machines are mostly FDM based for general and educational purposes, having limited applications in the healthcare sector. Therefore, there remains a tremendous growth potential for medical 3DP in Pakistan. Considering the wide array of possible uses, there is an appalling need for policymakers, public/private healthcare organizations to facilitate acquisition of this latest technology.
